# Comparison of Biodegradation of Nonylphenol Propoxylates with Usage of Two Different Sources of Activated Sludge

**DOI:** 10.1007/s11743-013-1537-2

**Published:** 2013-10-22

**Authors:** Agnieszka Zgoła-Grześkowiak, Tomasz Grześkowiak, Andrzej Szymański

**Affiliations:** Institute of Chemistry and Technical Electrochemistry, Poznan University of Technology, Piotrowo 3, 60-965 Poznan, Poland

**Keywords:** Nonylphenol propoxylates, Biodegradation, Municipal sewage treatment plant, Rural sewage treatment plant

## Abstract

Aerobic biodegradation behaviour of nonylphenol propoxylates was investigated in two tests with different sewage sludge as inocula. The samples containing target compounds were pre-concentrated using dispersive liquid–liquid microextraction and analysed with the use of high performance liquid chromatography with tandem mass spectrometry. Both primary biodegradation and formation of different biodegradation by-products were studied. Primary biodegradation of nonylphenol propoxylates was relatively slow and reached only about 70 % in over 70 days from the start of the tests. The biodegradation by-products from both oxidative and non-oxidative pathways were found. In the non-oxidative route, shortening of the propoxy chain was observed. In the oxidative pathway carboxylic acids and ketones were identified. The biodegradation by-products identified with the use of mass spectrometric detection also persisted for many days.

## Introduction

Alkoxylates are the dominant group of nonionic surfactants, both in terms of production volume and variety of applications. The most important of these are ethoxylates including alkylphenol ethoxylates (APEOs). Alkylphenol propoxylates (APPOs) have been known, commercially produced, and used for about 60 years. However, they have never been produced in such large quantities as APEOs.

In the last few years, the range of technological and industrial applications of APPOs has been greatly expanded. The first major technological application of APPOs [including nonylphenol propoxylates (NPPOs)], patented in the early 1970s, predicted their addition to fuel blends for automotive engines. By using a fuel composition containing a small but effective amount of APPOs, the hydrocarbon emission in the exhaust from an internal combustion engine is substantially reduced [1]. About 15 years ago, attention was drawn to the possibility of effective use of APPOs as novel plasticisers for epoxy resins and aminic epoxy resin hardeners [2]. APPOs may also be used as dispersants in pharmaceutical and agrochemical formulations [3]. Moreover, we can note the increased use of APPOs in the textile industry as dyeing assistants for dispersing dyes, because of the well-known suitability for this purpose of compounds with a similar structure to APPOs (containing a propoxylene-ethoxylene-propoxylene block copolymer chain connected to the alkylphenyl substituent) [4]. Because of their generally low polarity, APPOs are also used as a co-surfactant in coatings and printing ink compositions and in low-foaming wetting agents.

Due to their chemical structure, APPOs are considered as important components of industrial and municipal wastewater, because compounds containing the propoxylene moiety may be rather resistant to biodegradation. In the case of poly(propylene glycol)s, such a view has been presented for many years [5, 6], as the studies from the past few years confirm [7, 8]. Biodegradation of alkylphenyl substituents connected to the propoxylene chain is difficult and requires special conditions. Moreover, alkylphenols have been known for several years as hazardous pollutants with endocrine disruption activity [9–11]. Many biodegradation studies on APEOs have been carried out [12–18], while only one study concerning the biodegradation of APPOs is available [19]. Most studies on APEOs have proved that biodegradation of these surfactants occurs by shortening the ethoxylene chain, which leads to accumulation of alkylphenols and APEOs containing one, two or three ethoxylene units in the environment [5, 20–28]. On the other hand, two studies carried out in the last decade showed the possibility of the biodegradation of APEOs by the “central fission” mechanism, which leads straight to the alkylphenols and poly(ethylene glycol)s as intermediate products of the biodegradation [29, 30].

A recent study on NPPOs proved a different biodegradation of APPOs than APEOs. No shortening of the oxypropylene chain was observed during the entire test [19]. Nevertheless, formation of free nonylphenol could not be excluded even though only a limited increase of nonylphenol concentration was noted [19]. It is also not easy to establish a reasonable biodegradation pathway without nonylphenol as the intermediate product of the ultimate biodegradation. On the other hand, further studies on NPPOs are important because it would be desirable to find out whether the difference between biodegradation of NPPOs and APEOs is just compound-dependent.

This paper compares the biodegradation of NPPOs with the use of two different sources of activated sludge used for the tests. The first sample of activated sludge was taken from a large municipal sewage treatment plant (STP) and the second one was from a small rural STP. Both primary biodegradation of NPPOs and formation of nonylphenol as a biodegradation by-product are compared and discussed.

## Materials and Methods

### Reagents and Chemicals

A mixture of NPPOs with an average propoxylation degree of 10 was obtained from Sasol (Johannesburg, South Africa) as NONFIX 11011. Nonylphenol (NP) was purchased from Sigma-Aldrich (St. Louis, MO, USA). MS-grade and HPLC-gradient grade methanol and acetonitrile (ACN) were from Sigma-Aldrich. Water was prepared by reverse osmosis in a Demiwa system from Watek (Ledec nad Sazavou, The Czech Republic), followed by double distillation from a quartz apparatus. Only freshly distilled water was used.

Analytical grade tetrachloroethylene applied as the extracting solvent in the experiments was purchased from Merck (Darmstadt, Germany). Analytical grade ethanol used as the dispersing solvent was obtained from J.T. Baker (Deventer, The Netherlands). MS-grade ammonium formate and ammonium acetate were purchased from Sigma-Aldrich. All reagents used for preparation of the test medium and synthetic sewage were obtained from POCh (Gliwice, Poland).

### Biodegradation Study (Modified OECD Screening Test)

Static screening tests for ready biodegradability in aerobic conditions were performed according to the OECD method 301E (Modified OECD Screening Test) [31]. NPPOs at a concentration of 0.5 mg L^−1^ were applied in two tests. Activated sludge from two different STPs was used as an inoculum in the tests. The inoculum taken for the first test was from a large STP located in Poznań (the Central STP) which treats 100,000 m^3^ day^−1^ of sewage. The inoculum from a small rural STP located in Tarnowo Podgórne and treating about 3,000 m^3^ day^−1^ of sewage was taken for the second test. The medium used in the test consisted of mineral components [KH_2_PO_4_, K_2_HPO_4_, Na_2_HPO_4_·2H_2_O, NH_4_Cl, CaCl_2_, MgSO_4_·7H_2_O, FeCl_3_·6H_2_O, MnSO_4_·4H_2_O, H_3_BO_3_, ZnSO_4_·7H_2_O and (NH_4_)_6_Mo_7_O_24_] in appropriate concentrations [31]. The tests were performed in 200-mL glass bottles. One bottle was prepared for each experimental point. The biodegradation tests lasted for 72 days.

### Sample Preparation Procedure

The samples from the test were separated and pre-concentrated before HPLC-MS analysis using a previously described method [19]. Briefly, a 6-mL water sample was placed in a 15-mL glass test tube with a conical bottom. Then, 2.5 mL of ethanol (dispersing solvent) containing 60 μL of tetrachloroethylene (extracting solvent) was injected rapidly into the sample solution using a 2.5-mL syringe. In this step, the extraction solvent was dispersed into the aqueous sample as very fine droplets and a cloudy solution was formed in the test tube. Then, the mixture was centrifuged for 10 min at 4,500 rpm (1947 RCF). The dispersed fine particles of extraction phase were sedimented in the bottom of the test tube. The sedimented phase was withdrawn with a 100-μL micro-syringe. Tetrachloroethylene was evaporated from the extract with a gentle nitrogen purge at room temperature and the residue was reconstituted to 30 μL of methanol and injected into the HPLC column for analysis.

### HPLC-MS Analysis of NPPOs and Oxidative Biodegradation Products

The analysis was performed as described previously [19] with modifications enabling use of one procedure for the analysis of both NPPOs and their biodegradation products. Briefly, a chromatographic system (UltiMate 3000 RSLC; Dionex, Sunnyvale, CA, USA) was used. The 5-μL samples were injected into a phenyl-hexyl column (50 mm × 3 mm I.D.; 1.8 μm) from Agilent Technologies (Santa Clara, CA, USA). The mobile phase employed in the analysis consisted of 5 × 10^−3^ mol L^−1^ ammonium formate in water and methanol at a flow rate of 0.5 mL min^−1^ at 35 °C. Gradient elution was performed by linearly increasing the percentage of organic modifier from 70 to 95 % in 15 min and then it was maintained at 95 % for 10 min. A pre-run time of 4 min was done before the next injection. The chromatographic system was connected to the API 4000 QTRAP triple quadrupole mass spectrometer from AB Sciex (Foster City, CA, USA). The LC column effluent was directed to the electrospray ionization source (Turbo Ion Spray) which was operated in positive ion mode. The following settings for the ion source and mass spectrometer were used: curtain gas 10 psi, nebulizer gas 40 psi, auxiliary gas 40 psi, temperature 300 °C, ion spray voltage 4,500 V, and declustering potential 40 V. The ammonium adducts of the analytes were determined in a scan mode.

### HPLC-MS Analysis of NP

The analysis was performed as described previously [19]. The LC-MS system and analytical column were the same as these used in analysis of NPPOs. The 5-μL samples were injected into the analytical column maintained at 35 °C. The mobile phase employed in the analysis consisted of 5 × 10^−3^ mol L^−1^ ammonium acetate in water and ACN at a flow rate of 0.5 mL min^−1^. The following gradient was used: 0 min 60 % ACN; 3 min 60 % ACN; 5 min 95 % ACN; 8 min 100 % ACN. A pre-run time of 4 min was carried out before the next injection. The Turbo Ion Spray source of the mass spectrometer operated in negative ion mode. The following settings for the ion source and mass spectrometer were used: curtain gas 20 psi, nebulizer gas 40 psi, auxiliary gas 40 psi, temperature 480 °C, ion spray voltage −4,500 V, declustering potential −80 V, and collision gas set to medium. The dwell time for each mass transition detected in the selected reaction monitoring mode was set to 100 ms. The quantitative transition was from 219.3 to 133.3 m/z at collision energy set to −48 V and the confirmatory transition was from 219.3 to 147.3 m/z at collision energy set to −35 V.

## Results and Discussion

**Fig. 1:**
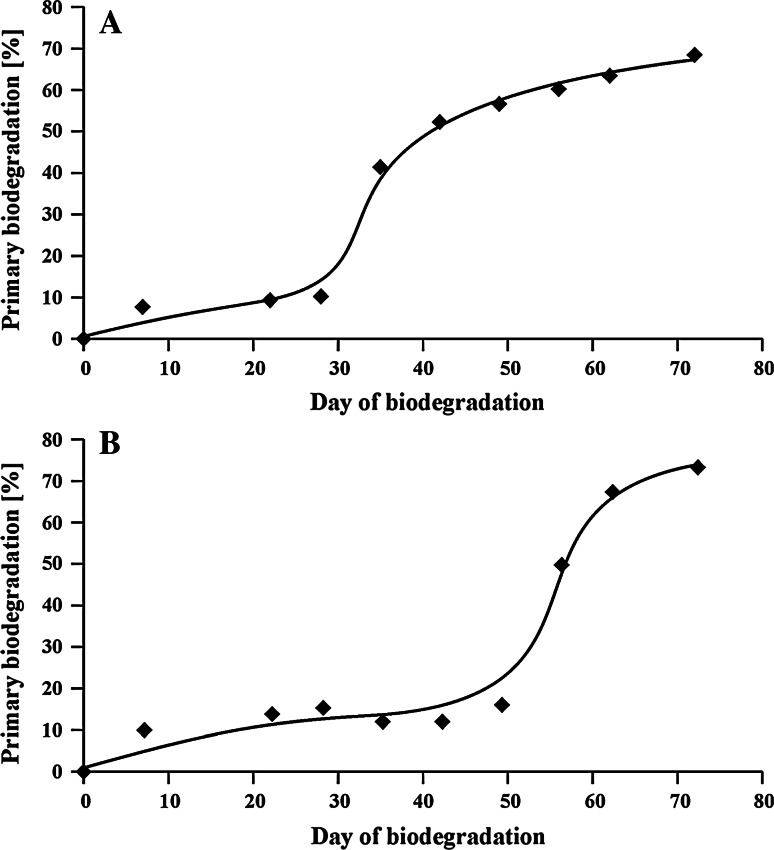
Primary biodegradation of nonylphenol propoxylates using inocula from **a** municipal STP, **b** rural STP

The biodegradation tests of NPPOs were made with the use of two different sewage sludge samples as inocula, which were taken from a large municipal STP (for test number one) and from a small rural STP (for test number two). The results gained in the tests show a rapid loss of about 10 % of NPPOs. This can be caused by unknown abiotic process, for example physical adsorption. Next, adaptation of microorganisms took place. This process was different in the two tests. Adaptation of microorganisms in sewage sludge taken from the Central STP to biodegradation of NPPOs was faster than these from the rural STP (Fig. 1). The difference was considerable—about 20 days. This could be caused by both different microbial strains existing in sewage sludge from the two STPs and higher amount of bacteria in sludge from the Central STP. Nevertheless, final primary biodegradation was similar in both tests reaching about 70 % in over 70 days. Complete primary biodegradation was not achieved, confirming a generally accepted view about difficult biodegradation of alkoxylated alkylphenols. This obviously has a serious impact on the condition of the environment.

**Fig. 2:**
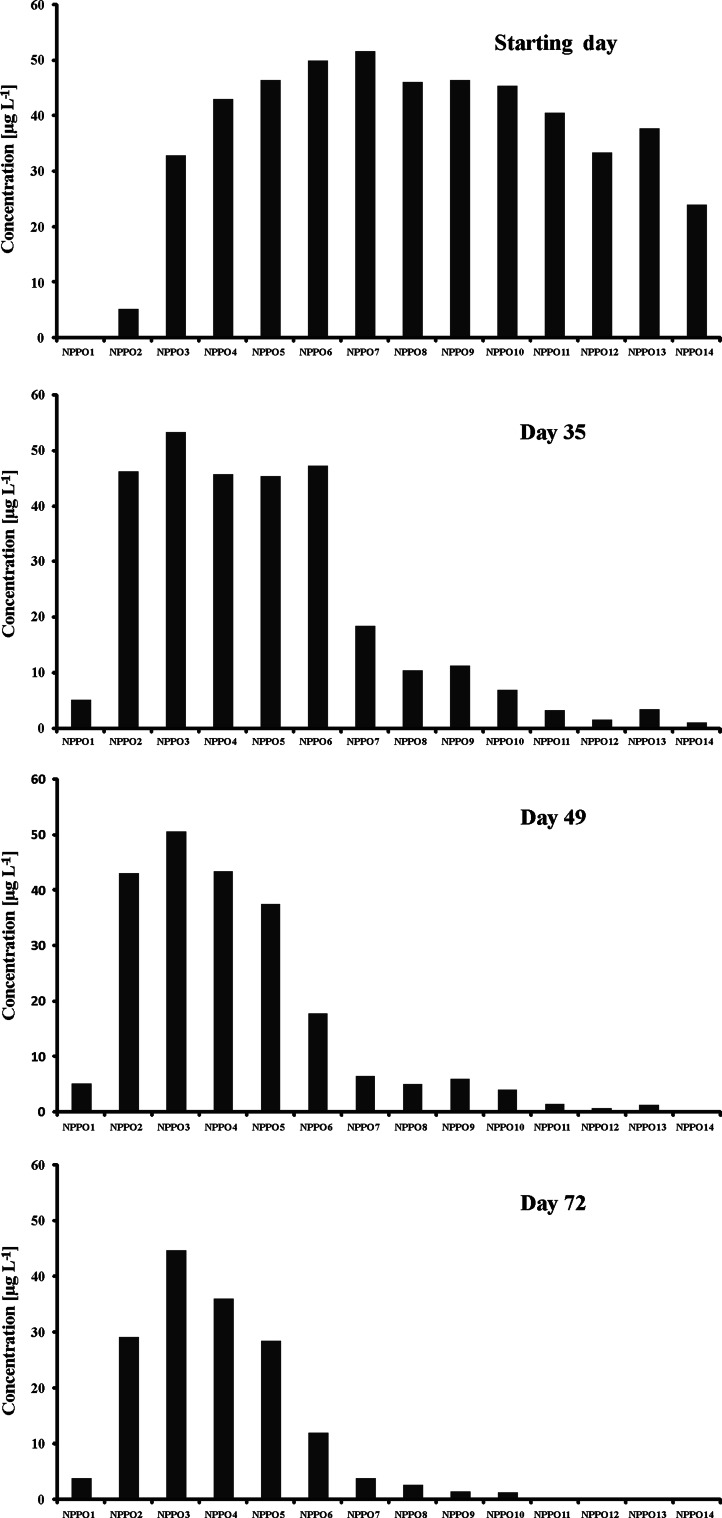
Profile of homologues of NPPOs during biodegradation using inoculum from municipal STP. Homologues containing 1–14 propoxy groups are designated with *NPPO1* to *NPPO14*, respectively

**Fig. 3:**
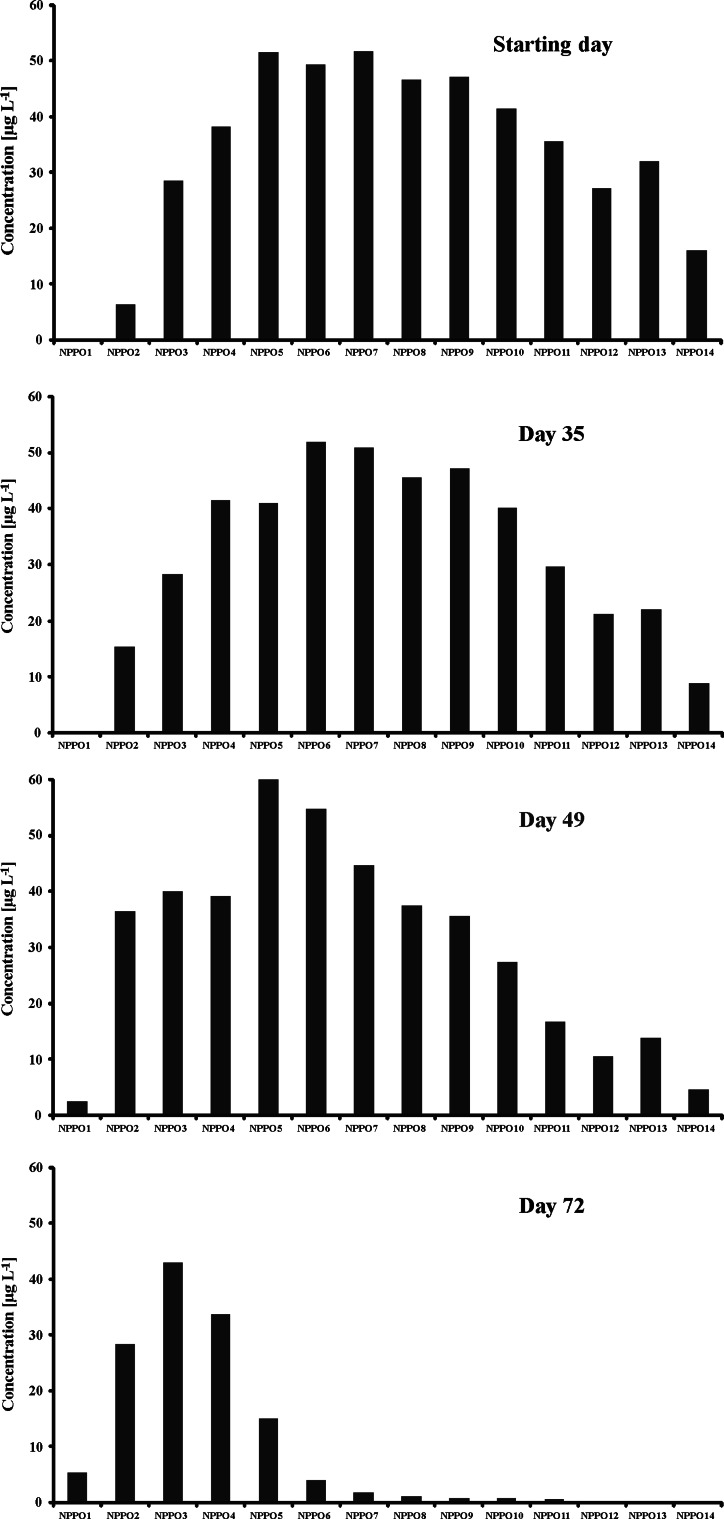
Profile of homologues of NPPOs during biodegradation using inoculum from rural STP. Homologues containing 1–14 propoxy groups are designated with *NPPO1* to *NPPO14*, respectively

The profiles of particular homologues of NPPOs recorded during the tests (Figs. 2, 3) changed. A step-by-step lowering of concentration of higher molecular mass homologues was noted in both tests which is characteristic of the domination of the propoxylene chain shortening mechanism. These results were surprising because no such considerable propoxylene chain shortening had been observed in our previous study on NPPOs [19]. Also, studies on biodegradation of poly(propylene glycol)s (PPGs) showed no propoxylene chain shortening mechanism [7, 8]. Instead, formation of acidic biodegradation products has been presented in previous studies on both NPPOs and PPGs [7, 8, 19]. The reason for the different biodegradation scheme can be most probably connected to the different bacteria present in the sewage sludge used in this study and the river water used previously [19]. It is well known that sewage directed to STPs contains considerable amounts of faecal bacteria. These bacteria are not completely removed during the biodegradation process carried out in STP [32]. Hence, considerable amounts of faecal bacteria including *Escherichia coli*, *Enterococcus faecalis*, *Pseudomonas aeruginosa* and others could be present in the biodegradation liquor used in this study. On the other hand, river water usually contains small amounts of faecal bacteria. As the faecal bacteria are mostly anaerobic [33], their presence could lead to the non-oxidative shortening of NPPO chains found in this study.

The rate of formation of lower molecular mass homologues of NPPOs was different in both tests, which is in accordance with the rate of primary biodegradation given in Fig. 1. However, the final profile of homologue distribution was similar in both tests (accumulation of NPPOs with one to six propoxylene units was observed in both cases) This scheme is similar to that observed in biodegradation tests of APEOs where lower molecular mass homologues having one to three ethoxylene units were accumulated [12, 15, 24]. The difference in alkoxylene chain lengths of NPPOs and APEOs being accumulated in the biodegradation liquor can be connected with the lower polarity of propoxylene units compared with ethoxylene units. As a result, the biodegradation slows down for compounds with similar nonpolar properties. On the other hand, formation of complexes of metal ions (present in biodegradation liquor) with short-chained NPPOs can be expected, similar to those described for short-chained APEOs [15]. Such stable metal complexes would diminish the biodegradation of lower molecular mass homologues of NPPOs. However, even limited biodegradation of short-chained NPPOs produces the lowest homologues, including monopropoxy nonylphenol. The very small abundance of its peak can be misleading, as its signal in mass spectrometry can be much lower than signals of higher molecular mass homologues of NPPOs. Similarly, monoethoxy alkylphenols give much lower abundances than higher homologues of APEOs at the same concentration [27, 28].

**Fig. 4:**
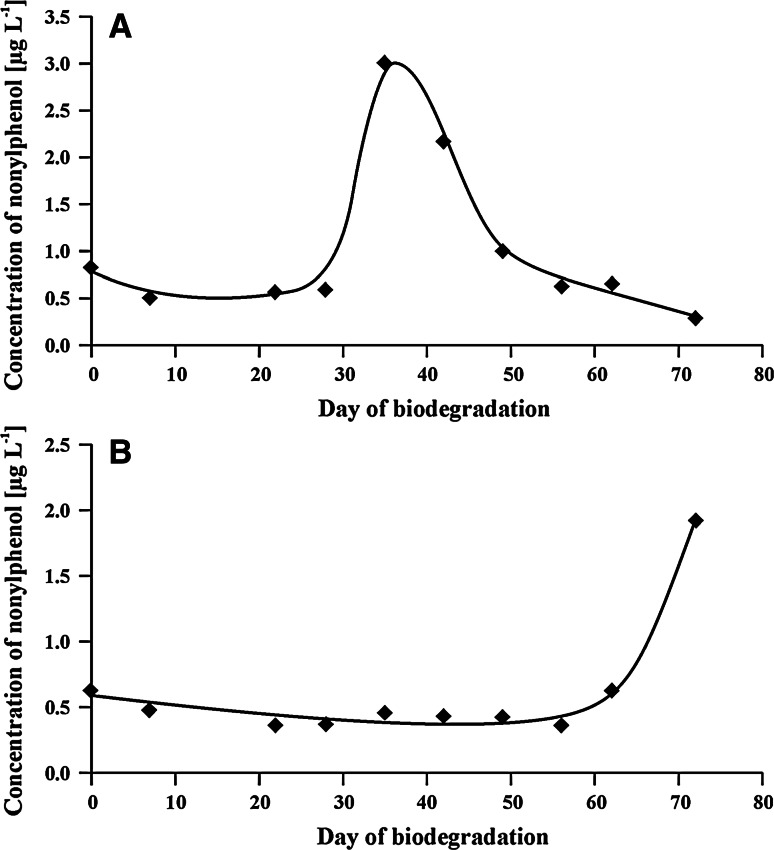
Concentration of nonylphenol during biodegradation of NPPOs using inocula from **a** municipal STP, **b** rural STP

Furthermore, formation of endocrine-disrupting nonylphenol was also observed during both biodegradation tests. The rapid increase of its concentration was noted on different days of the two tests (Fig. 4). However, this increase was correlated with the faster biodegradation rate of NPPOs in the first test than in the second one (Fig. 1). It is also worth stressing that NP formed in the test with the inoculum from the Central STP was biodegraded in about 2 weeks. No abiotic loss (e.g. adsorption) was considered in this process because NP was formed in a concentration about 1,000 times lower than its solubility in water. Biodegradation of NP was not observed in the second test, because the fast formation of NP was found only in the last days of this test. However, in analogy to the first test, a similar lowering of NP concentration can be expected. This can be further supported by the results presented in our previous study [19]. Also, comparison with the previously obtained results shows about three times higher concentrations of NP obtained in the present study with notable shortening of the propoxylene chain. Moreover, the fast biodegradation of NP (noted during the test with the inoculum from the Central STP) refutes the thesis that slow biodegradation of short-chained NPPOs is caused by their low polarity. On the contrary, these results suggest that formation of stable metal complexes by these compounds is the source of the lower biodegradation rate of short-chained NPPOs.

**Fig. 5:**
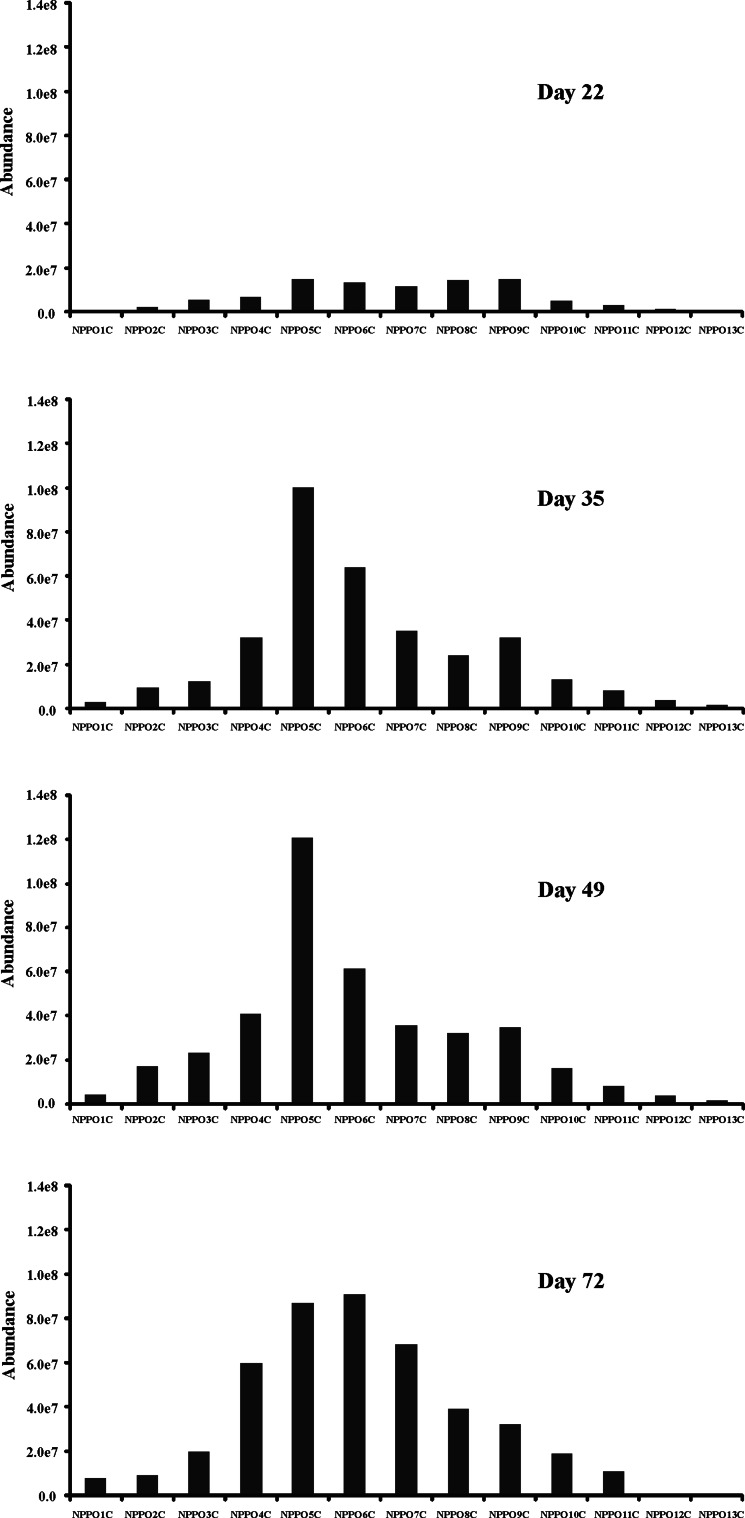
Profile of homologues of carboxylated biodegradation products of NPPOs during biodegradation using the inoculum from the municipal STP. Homologues containing 1–13 propoxy groups are designated with *NPPO1C* to *NPPO13C*, respectively

**Fig. 6:**
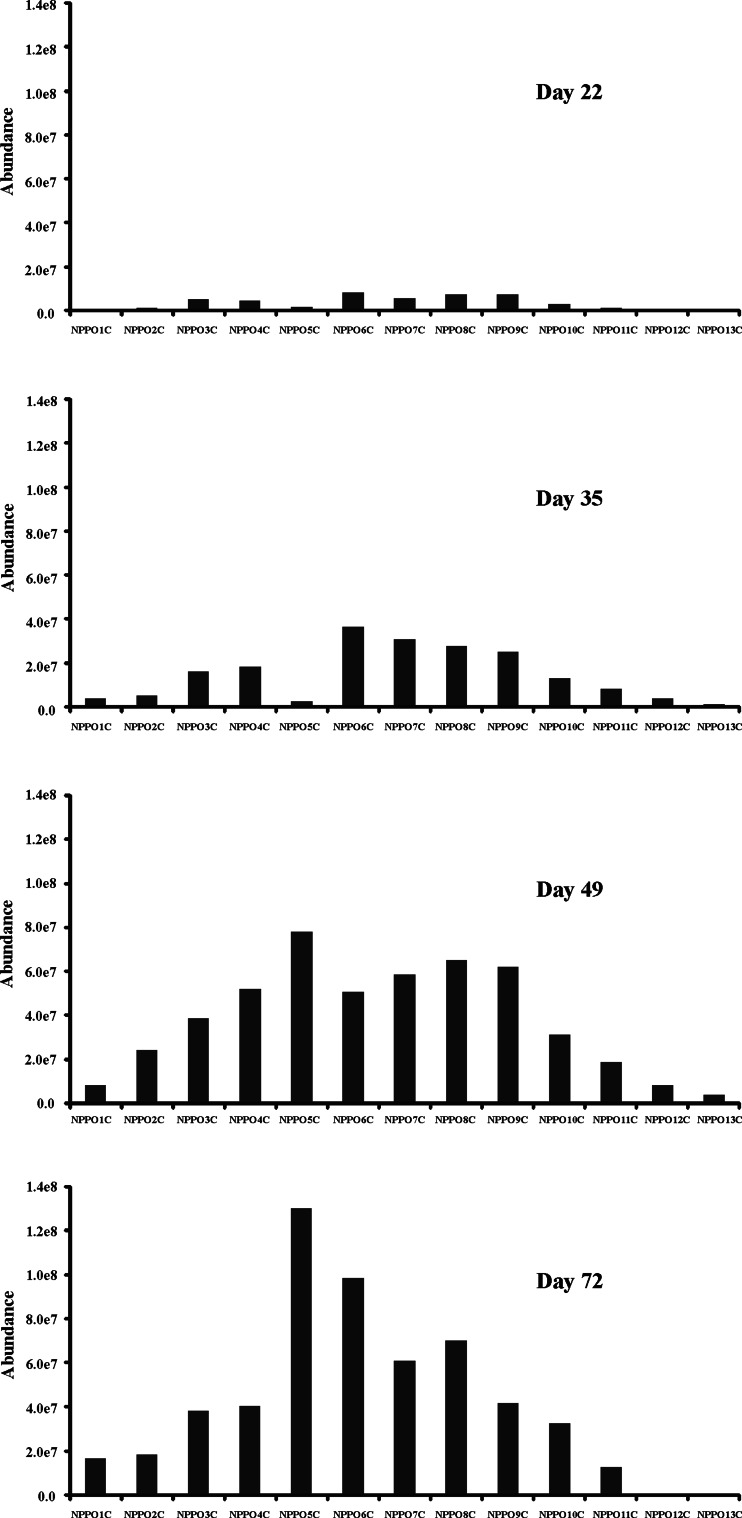
Profile of homologues of carboxylated biodegradation products of NPPOs during biodegradation using the inoculum from the rural STP. Homologues containing 1–13 propoxy groups are designated with *NPPO1C* to *NPPO13C*, respectively

The oxidative biodegradation of tested NPPOs was also monitored. The oxidative biodegradation products found in this study were analogous to these identified in our previous paper [19]. Formation of these products was already observed in both tests on the 22nd day of biodegradation (Figs. 5, 6), that is earlier than when any significant biodegradation of the parent NPPOs was observed. Higher amounts of carboxylic biodegradation products were found on the 35th day and they persisted to the end of both tests. Formation of carboxylic acids in the first test was found at a slightly higher level than in the second one, which is in accordance with the biodegradation rate of NPPOs. It is also worth stressing that no considerable shortening of propoxylene chains was observed in the oxidative biodegradation route and the formation of short-chained products was limited. Instead, the accumulation of carboxylic biodegradation products with an average of five to six propoxylene units was noted, i.e. about two propoxylene units more than was found for non-oxidative biodegradation products.

**Fig. 7:**
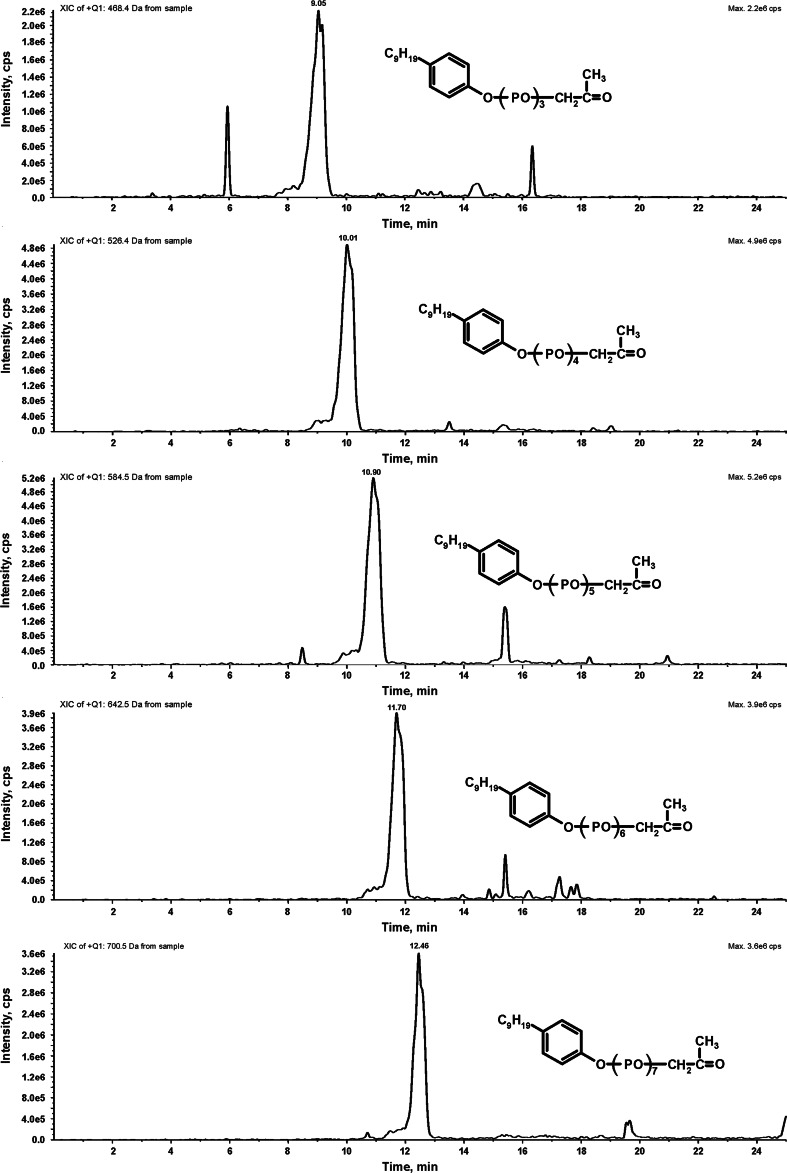
Extracted ion chromatograms of ketone biodegradation products found on the 35th day of biodegradation of NPPOs using the inoculum from the municipal STP. The ammonium adducts of ketones with 4–8 propoxy groups were found at *m*/*z* = 468, 526, 584, 642 and 700, respectively

Finally, traces of some ketone biodegradation products were found in the biodegradation liquors. Extracted ion chromatograms taken from a sample containing these biodegradation products are presented in Fig. 7. Formation of these compounds means that NPPOs contain not just head to tail configuration of propoxylene units. As a result, oxidative biodegradation of secondary alcohols formed during propoxylene chain scission led to ketones.

**Fig. 8:**
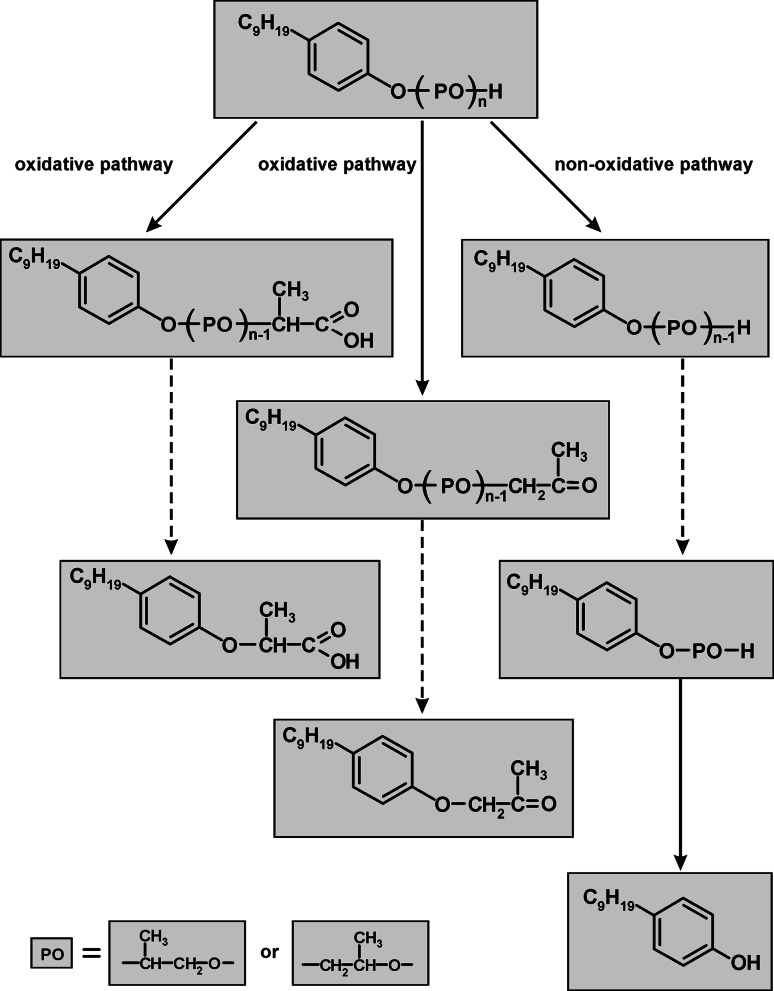
Proposed biodegradation scheme of NPPOs. For simplification connections between pathways are not included. These are possible, as not only head to tail configuration of propoxylene units exists but changes between oxidative and non-oxidative biodegradation can also be met

The proposed biodegradation pathways of NPPOs are presented in Fig. 8. Both oxidative and non-oxidative pathways can be included in this proposal. Nevertheless, no information can be given about the proportion of oxidative and non-oxidative biodegradation products formed in the tests, as there are no standards for these compounds. Further studies could be undertaken to identify the reason for the shortening of the propoxylene chains and, connected with it, the formation of higher amounts of NP.

## Conclusion

The biodegradation of NPPOs by microorganisms from two different samples of sewage sludge used as inocula (one sewage sludge sample was taken from a large municipal STP, the other from a small rural STP) was studied. Static screening tests for ready biodegradability were performed in aerobic conditions, according to the Modified OECD Screening Test 301E.

Although the time of adaptation to NPPOs biodegradation by microorganisms from the municipal wastewater treatment plant was about 20 days shorter than for micro-organisms from rural STP, the final primary biodegradation was similar in both cases, reaching about 70 % in over 70 days. Systematic lowering of concentration of homologues containing a long propoxylene chain, and accumulation of homologues containing a short propoxylene chain in the biodegradation liquor was noted in both tests, which is characteristic of the domination of the propoxylene chain shortening mechanism.

The formation of noticeable amounts of endocrine-disrupting nonylphenol and large amounts of carboxylic biodegradation by-products was also observed during both biodegradation tests. The presence of these biodegradation by-products suggests that the mechanisms of APPOs biodegradation are largely consistent with the much better studied mechanisms of APEOs biodegradation.

## References

[1] Manary OJ (to Dow Chemical Company) (1971) Gasoline fuel containing polyalkoxylated alkylphenol to reduce exhaust emission. US Patent 3,615,295

[2] Scherzer W, Volle J (to Ciba Spezialitätenchemie Bergkamen GMBH) (1999) Propoxylated phenols and/or propoxylated aromatic alcohols as plasticisers for epoxy resins and aminic epoxy resin hardeners. International Application published under the Patent Cooperation Treaty, PCT/EP99/00280

[3] Meier HM, Knackmuss HJ, Rieger PG (to Bayer Aktiengesellschaft) (2003) Verwendung alkoksylierter phenolderivate. Europäische Patentanmeldung EP 1 285 941 A1

[4] Heller J (to Sandoz Ltd.) (1984) Use of polyoxyalkylated alkyl phenols as dyeing assistants for disperse dyes. US Patent 4,490,150

[5] Swisher RD (1987). Surfactants biodegradation.

[6] Balson T, Felix MSB, Karasa DR, Porter MR (1995). Biodegradability of non-ionic surfactants. Biodegradability of Surfactants.

[7] Zgoła-Grześkowiak A, Grześkowiak T, Zembrzuska J, Łukaszewski Z (2006). Comparison of biodegradation of poly(ethylene glycol)s and poly(propylene glycol)s. Chemosphere.

[8] Zgoła-Grześkowiak A, Grześkowiak T, Zembrzuska J, Frańska M, Frański R, Kozik T, Łukaszewski Z (2007). Biodegradation of poly(propylene glycol)s under the conditions of the OECD screening test. Chemosphere.

[9] Jobling S, Sumpter JP (1993). Detergent components in sewage effluents are weakly oestrogenic to fish: an in vitro study using rainbow trout *(Oncorhynchus mykiss)* hepatocytes. Aquat Toxicol.

[10] White R, Jobling S, Hoare SA, Sumpter JP, Parker MG (1994). Environmentally persistent alkylphenolic compounds are estrogenic. Endocrinology.

[11] Soto AM, Sonnenschein C, Chung KL, Fernandez MF, Olea N, Serrano FO (1995). The E-screen assay as a tool to identify estrogens: an update on estrogenic environmental pollutants. Environ Health Persp.

[12] Maki H, Masuda N, Fujiwara Y, Ike M, Fujita M (1994). Degradation of alkylphenol ethoxylates by *Pseudomonas* sp. strain TR01. Appl Environ Microbiol.

[13] Staples CA, Naylor CG, Williams JB, Gledhill WE (2001). Ultimate biodegradation of alkylphenol ethoxylate surfactants and their biodegradation intermediates. Environ Toxicol Chem.

[14] Langford KH, Scrimshaw MD, Birkett JW, Lester JN (2005). Degradation of nonylphenolic surfactants in activated sludge batch tests. Wat Res.

[15] Frańska M, Ginter-Kramarczyk D, Szymański A, Kozik T, Frański R (2009). Resistance of alkylphenol ethoxylate containing six ethoxylene units to biodegradation under the conditions of OECD (Organization for Economic Co-operation and Development) screening test. Int Biodeter Biodeg.

[16] Zhao J, Zhang G, Qin Y, Zhao Y (2006). Aerobic biodegradation of alkylphenol ethoxylates. Bioresour Technol.

[17] Hayashi S, Saito S, Kim J-H, Nishimura O, Sudo R (2005). Aerobic biodegradation behaviour of nonylphenol polyethoxylates and their metabolites in the presence of organic matter. Environ Sci Technol.

[18] Jonkers N, Knepper TP, de Voogt P (2001). Aerobic biodegradation studies of nonylphenol ethoxylates in river water using liquid chromatography-electrospray tandem mass spectrometry. Environ Sci Technol.

[19] Zgoła-Grześkowiak A Development of a dispersive liquid-liquid microextraction procedure for biodegradation studies on nonylphenol propoxylates under aerobic conditions. J Surfact Deterg. doi:10.1007/s11743-013-1479-8 10.1007/s11743-013-1479-8 PMC3880491 24415899

[20] Ahel M, Giger W, Koch M (1994). Behaviour of alkylphenol polyethoxylate surfactants in the aquatic environment - 1. Occurrence and transformation in sewage treatment. Wat Res.

[21] Zgoła-Grześkowiak A, Grześkowiak T (2009). Liquid chromatography with fluorescence detection as a tool for separation of endocrine disrupting alkylphenols and their mono- and diethoxylates in analysis of river water samples. Tenside Surf Deterg.

[22] Zgoła-Grześkowiak A, Grześkowiak T, Rydlichowski R, Łukaszewski Z (2010). Concentrations of endocrine disrupting alkylphenols and their mono- and diethoxylates in sediments and water from artificial Lake Malta in Poland. Tenside Surf Deterg.

[23] Zgoła-Grześkowiak A, Grześkowiak T (2011). Determination of alkylphenols and their short-chained ethoxylates in Polish river waters. Int J Environ Anal Chem.

[24] Zgoła-Grześkowiak A, Grześkowiak T, Rydlichowski R, Hołderna-Odachowska A, Łukaszewski Z (2011). The use of polytetrafluoroethylene multi-capillary trap extraction for isolation of octylphenol and its short-chained oxyethylates from the water matrix. J Chrom Sci.

[25] Heemken OP, Reincke H, Stachel B, Theobald N (2001). The occurence of xenoestrogens in the Elbe river and the North Sea. Chemosphere.

[26] Ferguson PL, Iden CR, Brownawell BJ (2001). Distribution and fate of neutral alkylphenol ethoxylate metabolites in a sewage-impacted urban estuary. Environ Sci Technol.

[27] Loyo-Rosales JE, Schmitz-Afonso I, Rice CP, Torrents A (2003). Analysis of octyl- and nonylphenol and their ethoxylates in water and sediments by liquid chromatography/tandem mass spectrometry. Anal Chem.

[28] Loos R, Hanke G, Umlauf G, Eisenreich SJ (2007). LC-MS-MS analysis and occurence of octyl- and nonylphenol, their ethoxylates and their carboxylates in Belgian and Italian textile industry, waste water treatment plant effluents and surface waters. Chemosphere.

[29] Frańska M, Frański R, Szymański A, Łukaszewski Z (2003). A central fission pathway in alkylphenol ethoxylate biodegradation. Wat Res.

[30] Wyrwas B, Chrzanowski Ł, Ławniczak Ł, Szulc A, Cyplik P, Białas W, Szymański A, Hołderna-Odachowska A (2011). Utilization of Triton X-100 and polyethylene glycols during surfactant-mediated biodegradation of diesel fuel. J Hazard Mater.

[31] OECD Guideline for testing of chemicals. Ready biodegradability (1992) Organisation for Economic Co-operation and Development, Adopted by the Council on 17 July 1992

[32] George I, Crop P, Servais P (2002). Fecal coliform removal in wastewater treatment plants studied by plate counts and enzymatic methods. Water Res.

[33] Wilhelm MP, Lee DT, Rosenblatt JE (1987). Bacterial interference by anaerobic species isolated from human feces. Eur J ClinMicobiol.

